# Self-Limited Kleptomania Symptoms as a Side Effect of Duloxetine

**DOI:** 10.1155/2016/5472492

**Published:** 2016-11-27

**Authors:** Christopher W. T. Miller, Keith E. Gallagher

**Affiliations:** ^1^University of Maryland School of Medicine, Baltimore, MD, USA; ^2^Sheppard Pratt Health System, Baltimore, MD, USA

## Abstract

*Introduction*. Impulse control disorders (ICDs) have been described as a side effect of dopamine agonists, frequently used in neurodegenerative conditions affecting the nigrostriatal pathway. Serotonin-norepinephrine reuptake inhibitors (e.g., duloxetine) have dose-dependent differential affinity for monoaminergic transporters, inhibiting the dopamine transporter at higher doses, thus increasing availability of synaptic dopamine, with the potential for similar impulse control side effects.* Case Presentation*. A 19-year-old Asian-American female with a history of depression developed new-onset stealing behaviors after an increase in her dose of duloxetine from 60 mg to 90 mg; she described these actions as “compulsive” and irresistible, later experiencing either relief or guilt, features compatible with an ICD. Her symptoms eventually subsided with continued use of 90 mg of duloxetine.* Discussion*. To the knowledge of the authors, this is the first report of a patient developing new-onset ICD behaviors after being placed on a higher dose of duloxetine, which can inhibit the dopamine transporter and cause difficulty with impulse control. The self-resolving nature of the symptoms may result from compensatory upregulation of dopamine transporters, increasing reuptake of dopamine. Asian populations may be at a higher risk due to the frequent occurrence of CYP2D6 polymorphisms, which decrease the conversion of duloxetine to its inactive metabolites.

## 1. Introduction

Impulse control disorders (ICDs) have been described as a side effect of agents with dopamine agonist activity. In particular, this has been noted in Parkinson's disease [1, 2], for which treatment often involves the addition of dopamine agonists to attempt to mitigate the hypodopaminergic state caused by degeneration of nigrostriatal dopamine neurons. However, indiscriminately elevating dopaminergic tone in the different projection areas of the substantia nigra causes differential effects anatomically: the ventral area leads to motor effects as it projects to the caudate nucleus and putamen, while the dorsal area leads to increased reward-driven behaviors and lower impulse control since projections are directed towards more behavior-oriented portions of the forebrain and limbic system [3].

One of the main classes of antidepressants in clinical use is the serotonin-norepinephrine reuptake inhibitors (e.g., duloxetine, venlafaxine, and milnacipran), which can display dose-dependent activity at different transporters, affecting the serotonin transporter (SERT) at lower doses, the norepinephrine transporter (NET) at intermediate doses, and the dopamine transporter (DAT) at higher doses [4]. As such, dose-dependent side effects may emerge as a result of this differential mechanistic property (e.g., the increase in diastolic blood pressure was observed as doses increase). While the effect at the DAT is not typically that pronounced, the emergence of clinical effects resulting from transporter blockade cannot be ruled out and could potentially simulate the dopamine agonistic effect described above. Both duloxetine and venlafaxine have reportedly caused impulsivity as a side effect, and “impulse control difficulties” are listed as a rare side effect of venlafaxine [5] but are not mentioned with duloxetine [6].

This report discusses the development of symptoms of kleptomania in a female patient which occurred after her dose of duloxetine was increased, which may speak to the effects of higher doses on the dopamine system.

## 2. Case Presentation

A 19-year-old Asian-American female with a reported history of unspecified depressive disorder and an eating disorder presented to the psychiatric emergency department with a one-month history of new-onset stealing behaviors. She described these as “compulsions” to steal a number of different items from her place of work (e.g., food items and pens); she described varying feelings of guilt or relief after engaging in these activities, and her presentation to the emergency room had been prompted by her actions being caught on a security camera, with her consequent dismissal from her position. As a result, she had developed acute suicidality, though without clear intent or plan, as she felt she was having these destructive urges which she could not understand or control. The patient denied any past impulse control behaviors of such a nature. She also reported multiple new financial stressors related to her college tuition and her family's finances but denied any conscious relationship of these stressors to her stealing. She denied any significant history of substance use disorders. The patient had no history suggestive of personality pathology; she denied any self-injurious behaviors (other than that related to her eating disorder) or suicide attempts; she denied patterns of affective instability, fear of rejection or abandonment, or past impulsive behaviors. There did not seem to be a history of periods of impaired reality testing nor of periods of paranoia or dissociation. There was no legal history whatsoever in the past, and the patient had maintained interpersonal, work, and academic functionality up until the current sequence of events. There was no indication that the patient had any secondary gain motivations to her presentation. No family history was available as she had been adopted at a very young age. Her eating disorder had begun around the age of 16 years and consisted of periods of restricting and times during which she would binge and purge. At the time of presentation, the patient's body mass index (BMI) was 15.88 kg/m^2^. Given her more internalizing traits, ruminating/obsessive thoughts about “being thin,” and her lack of impulsivity in other arenas, it was felt that her working DSM-5 eating disorder diagnosis was anorexia nervosa—binge-eating/purging type—which is less associated with more impulsive and externalizing personality traits than bulimia nervosa. Her depressive symptoms were qualified as mainly periods of dysphoria, negative ruminations about self, and passive death wishes. The patient had been started some years before on fluoxetine for a few months (unknown dosage) without much of a response. More recently, she had been started on duloxetine for the past several months, the dose being titrated up to 60 mg daily. As the patient was still complaining of lingering depressive symptoms, her primary physician increased the dose to 90 mg daily. Very soon after this increase, the patient's stealing behaviors began and persisted for four weeks, leading up to her presentation to the emergency room. Given the correlation of her stealing behavior with an increase in her duloxetine dose, a recommendation was made to decrease her dose back down to 60 mg and follow up with her outpatient provider. The patient was contacted again for follow-up two months later. At that time, her stealing behaviors had resolved despite having continued to take 90 mg of duloxetine daily. No additional medications had been added and there was no psychotherapeutic intervention implemented in the ensuing time.

## 3. Discussion

To the knowledge of the authors, ICDs have not been previously described as a side effect of duloxetine. In fact, serotonergic medications have been among the primary pharmacological agents used to treat ICDs [7, 8]. This case illustrates the development of an ICD as a result of an increase in the dosage of duloxetine, which, as mentioned, can lead to elevated dopaminergic tone due to DAT inhibition. Increased availability of dopamine can lead to increased agonism of D2 and D3 receptors in limbic areas [3], which can in turn diminish the control an individual has over self-governed risk-taking behaviors and enhance activities aimed at hedonic responses.

Though genotyping of this patient was not available, polymorphisms of the P450 isozyme 2D6 have been described in Asian populations and may result in low-functioning enzymatic activity (e.g., the CYP2D6^*∗*^10 polymorphism has been detected in up to 50% of some Asian populations) [9]. In patients taking concurrent 2D6 blockers, duloxetine levels can increase up to 60%; thus, a hypofunctioning enzyme can considerably impact metabolism [6]. Given that the clinical effect of duloxetine depends upon the availability of the parent drug (metabolites deriving from oxidation and subsequent conjugation are clinically inactive) and that metabolism of this drug is driven mainly by 2D6, lower activity of the latter could increase the likelihood of activity at the DAT, which supports the argument of this report. Body habitus itself may influence the concentrations of duloxetine and increase blood levels. One study showed that, compared with Caucasians, Japanese individuals had a maximum serum concentration (*C*_max_) around 20% greater; this was felt to be due to lower average body weight of Japanese subjects [10]; this patient's BMI would classify her as underweight and potentially increase drug levels.

The self-limited nature of this side effect (given that she remained at the same dose) may be explained by the effect chronic antidepressant use has on transporter availability. A single-photon emission computed tomography (SPECT) study by Kugaya et al. offers an intriguing mechanism [11]. In this study, chronic use of citalopram was noted to cause a decrease in SERT binding by a radioligand, yet there was an increase in availability of DAT. Chronic antidepressant use can lead to desensitization of serotonin receptors on dopaminergic neurons and can also lead to an increase in DAT, thus decreasing the availability of synaptic dopamine [11, 12]. This could account for the initial increase in dopamine availability through DAT inhibition with duloxetine, thus leading to impulse control issues, and subsequent normalization of dopamine levels through upregulation of DAT (even at the same dose), with consequent disappearance of the behaviors.

In conclusion, though ICDs are more typically a side effect of dopamine agonist drugs, attention should also be given to the possibility of DAT inhibitors causing a similar clinical effect, as there is also an observable increase in dopaminergic tone which could potentially favor a more reward-driven behavior profile. Given the high inhibitor constant (Ki) of duloxetine for the DAT (around 230 nmol/L) [13], which is less robust than its affinity for SERT and NET, there is certainly the possibility that the behaviors were coincidental. It should be mentioned that this Ki does not rule out a clinical effect; as a comparison, the Ki of venlafaxine for NET is 1920 nmol/L [13], and its noradrenergic effect factors into its therapeutic properties. The close proximity between the increase in dose and development of these new behaviors in this patient, particularly with no evidence of premorbid personality pathology suggestive of trait impulsivity, raises the possibility of this phenomenon being medication-induced. Future studies could help elucidate the association posited by this case report between duloxetine and ICDs.
